# Psychological and clinical characteristics of female patients with spontaneous coronary artery dissection

**DOI:** 10.1007/s12471-020-01437-7

**Published:** 2020-06-04

**Authors:** V. R. Smaardijk, P. M. C. Mommersteeg, W. J. Kop, D. Adlam, A. H. E. M. Maas

**Affiliations:** 1 Department of Medical and Clinical Psychology, Centre of Research on Psychology in Somatic Diseases (CoRPS), Tilburg University, Tilburg, The Netherlands; 2 Department of Cardiovascular Sciences and Leicester NIHR Cardiovascular Biomedical Research Centre, University of Leicester, Leicester, UK; 3 Department of Cardiology, Radboud University Medical Centre, Nijmegen, The Netherlands

**Keywords:** Spontaneous coronary artery dissection, Women, Myocardial infarction, Risk factors

## Abstract

**Aims:**

Spontaneous coronary artery dissection (SCAD) is increasingly recognised as a cause of myocardial infarction, but psychological characteristics of patients with SCAD have not yet been extensively investigated. We assessed the prevalence of a broad range of psychological and clinical factors, and their inter-relationships in patients with a history of SCAD. Furthermore, we investigated whether specific clusters of patients with SCAD can be identified.

**Methods:**

Participants were recruited between March and May 2019 from a Dutch SCAD database and completed online questionnaires. Clinical information was verified by review of medical records. Participants were predominantly female (172/183; 94%). Analyses focused on the 172 female patients (mean age 52.0 ± 7.5 years, 37% postmenopausal).

**Results:**

The most common comorbidities of SCAD were migraine (52%), fibromuscular dysplasia (FMD; 29%), chronic pain (29%), and tinnitus (28%). Six women (3%) had pregnancy-associated SCAD. Traditional cardiovascular risk factors were rare (<10%), except for hypertension (31%). Psychological assessment indicated high levels of perceived stress (PSS-10 ≥14; 50%), fatigue (FAS-10 ≥22; 56%), and a frequent history of burnout (25%). The prevalence of depression (9%) and anxiety (12%) was relatively low. Three clusters were identified: (A) FMD and chronic non-ischaemic conditions (tinnitus, chronic pain, and irritable bowel syndrome); (B) migraine; and (C) none of these conditions.

**Conclusion:**

This study shows that perceived stress and fatigue are common in patients with SCAD, in addition to prevalent comorbid FMD, migraine, tinnitus, and non-ischaemic pain conditions. These factors may add to developing tailored rehabilitation programmes for patients with SCAD.

**Electronic supplementary material:**

The online version of this article (10.1007/s12471-020-01437-7) contains supplementary material, which is available to authorized users.

## What’s new?

- Patients with a history of spontaneous coronary artery dissection (SCAD) have high levels of perceived stress and fatigue, whereas levels of anxiety and depression are relatively low.
- Several chronic conditions that do not reflect ischaemic or non-ischaemic cardiovascular disease (including tinnitus, chronic pain and burnout) are frequently reported precursors or comorbidities of SCAD.
- Psychological factors, in addition to fibromuscular dysplasia, migraine, tinnitus, and non-ischaemic pain conditions, may contribute to the development of SCAD and its subsequent clinical course.

## Introduction

Spontaneous coronary artery dissection (SCAD) involves sudden tearing of the intima of the vessel wall in one of the coronary arteries, resulting in myocardial ischaemia or infarction [1]. Traditional cardiovascular risk factors are less prominent in the development of SCAD [2, 3]. In women with acute coronary syndrome (ACS) below 60 years of age, the estimated prevalence of SCAD ranges between 23 and 36%, whereas SCAD is much less common (less than 10%) in men [1].

The pathophysiology of SCAD is heterogeneous and remains insufficiently defined. Differences in sex hormones may play an important role, as well as genetic factors, pregnancy status, and comorbidities such as connective tissue diseases and fibromuscular dysplasia (FMD) [1, 2]. About 42% of SCAD patients present with (a history of) migraine [4].

Symptoms of anxiety and depression are common in patients with SCAD, especially in younger women and in those with peripartum SCAD [5]. However, other psychological characteristics in SCAD patients have not been documented. Conditions reflecting psychological distress, such as anxiety and depression, develop in the context of psychosocial factors, including personality characteristics and patients’ reactions to physical symptoms [6]. In this study, we assess the prevalence of psychological factors and clinical characteristics in patients with a history of SCAD and investigate whether specific clusters of SCAD patients can be identified. Results from this study may help understand the long-term psychological consequences and symptom burden in patients who experienced SCAD. This information may lead to individually tailored rehabilitation, clinical follow-up, and psychological support strategies.

## Methods

### Participants and setting

Patients with SCAD were recruited between March and May 2019 from the Radboud University Medical Centre (Radboudumc, Nijmegen, The Netherlands) SCAD database. This database is specifically designed to provide a knowledge platform for SCAD patients from the Netherlands and involves inclusion from tertiary referral outpatient clinics as well as self-registration.

In order to be included in this study, participants had to be diagnosed with SCAD based on coronary angiographic assessments [7] and have sufficient knowledge of the Dutch language. Patients with atherosclerotic, traumatic or iatrogenic SCAD were excluded. The research protocol was approved by the local research ethics board (#2018-5017).

### Procedure

Eligible participants were contacted via e‑mail and were asked to provide digital informed consent to examine their hospital records as well as to fill out web-based questionnaires. This informed consent was provided via an online data capture platform (www.castoredc.com). Subsequently, an online questionnaire including demographic, clinical, women-specific, and psychological factors was sent to those who gave permission. An additional questionnaire and a request to send a letter from their cardiologist who confirmed the SCAD diagnosis based on coronary angiography was sent to participants without a medical record at the Radboudumc.

A total of 232 (216 female and 16 male) potential patients with SCAD were approached, of whom 219 (94%) responded. Of these, 210 gave permission to participate, and 172 women and 11 men with confirmed SCAD diagnoses completed the questionnaires. As a consequence of the low prevalence of male SCAD patients, these were included only in our explorative group comparisons.

### Measures

#### SCAD-related characteristics

We investigated age at the (most recent) SCAD event, pregnancy-associated SCAD (P-SCAD), and whether multiple SCADs or other types of ACS events occurred. We also calculated the time between SCAD and survey completion.

#### Psychological factors

The psychological factors investigated in this study were based on the screening guidelines for psychological risk factors for cardiovascular disease as developed by the European Society of Cardiology [8].

The 7‑item General Anxiety Disorder questionnaire (GAD-7) was used as a measure of anxiety [9]. Participants rated how often in the past 2 weeks they were bothered by specific symptoms. A previously validated cutoff of 10 was used to identify persons with moderate or severe symptoms of anxiety.

Depressive symptoms were assessed using the Patient Health Questionnaire (PHQ-9) [10]. The items inquire about specific symptoms of depression over the past 2 weeks. A previously validated cutoff of 10 was used to identify persons with moderate or severe depressive symptoms.

The Perceived Stress Scale (PSS) was used to measure the severity of psychological distress in the past month [11]. Compared to the original 14-item PSS, the 10-item version shows better psychometric characteristics and was used in the present study [12]. A cutoff of 14 was used to identify participants with moderate or high perceived stress.

The Fatigue Assessment Scale (FAS-10) was used to measure symptoms of chronic fatigue. Participants reported on 10 items how they usually feel [13]. A cutoff of 22 was used to assess substantial or extreme fatigue [14]. We assessed the attentional focus on body sensations with the Body Vigilance Scale (BVS-3) [15]. Positive mental well-being in the past month was assessed with the Mental Health Continuum-Short Form (MHC-SF) [16]. This instrument consists of 14 items and assesses emotional, psychological, and social well-being.

Type D personality was assessed using the Type D Scale (DS14). This instrument contains 7‑item negative affectivity (NA) and social inhibition (SI) subscales [17]. A cutoff of 10 (NA ≥10 and SI ≥10) was used to identify Type D [17]. Neuroticism was assessed with 8 items from the Dutch version of the Big Five Inventory (BFI-NL) [18]. Mean normative values are 2.6 for people aged 40–60 years [18].

#### Clinical characteristics and covariates

Information about clinical characteristics and demographic factors was obtained from medical records and online self-reported questionnaires. For each comorbid clinical condition, participants reported whether the disorder was currently present, or in the past, or not present at all.

With regard to FMD, data were double-checked using medical records. The presence of FMD was based on angiographic testing of the renal arteries and/or other vascular beds. With regard to migraine, we inquired about the current presence and history of the condition using the participants’ self-reported data. Except for the presence of burnout, comorbidities currently present or in the past were combined in our analyses.

### Statistical analyses

Descriptive statistics are provided for participant characteristics with continuous variables reported as mean ± standard deviation (SD) or interquartile range, and categorical variables as frequencies and percentages.

To determine whether specific subgroups of SCAD patients could be identified, we conducted hierarchical cluster analyses. Clusters were identified using the Ward method [19] with squared Euclidean distances (values were standardised from −1 to +1 to make distances scale-independent). Selection of the number of clusters was based on inspection of the distance coefficient curves.

Additionally, we assessed group differences on presence/absence of FMD, presence/absence of migraine, perceived stress level, and sex. Categorical variables were compared with Chi-square tests or Fisher’s exact tests. Continuous variables were compared with ANOVA. A *p*-value below 0.05 was considered statistically significant. Statistical analyses were performed using SPSS (version 24).

## Results

### Participant characteristics

The mean age at the most recent SCAD was 49.4 ± 7.6 years. Six participants (3%) had a SCAD during their pregnancy or within 2 years of delivery (P-SCAD). The median time between SCAD and survey completion was 2.4 years.

Tab. 1 shows the characteristics of the 172 female patients with SCAD. The mean age at the time of study was 52.0 ± 7.5 years. The prevalence of traditional cardiovascular risk factors was low (<10%), with the exception of hypertension (31%). Twenty percent were on hormonal therapy.

**Table 1:** Characteristics of female patients with spontaneous coronary artery dissection (*n* = 172)

Characteristics	Women (*n* = 172)^a^
*Demographic factors*	
Age (years)	52.0 ± 7.5
European descent	169 (98%)
Married or in a relationship	154 (90%)
Employment status	
– Employed, working full time	31 (18%)
– Employed, working part time	98 (57%)
– Other (e.g. unemployed, retired, homemaker)	43 (25%)
Education level	
– University education or higher professional education	96 (56%)
– Secondary vocational education	62 (36%)
– Secondary education	12 (7%)
– Primary education	2 (1%)
*Cardiovascular risk factors*	
Hypertension	54 (31%)
Hypercholesterolaemia	15 (9%)
Diabetes mellitus	2 (1%)
Family history of heart disease <60 years	80 (47%)
Current smoker	4 (2%)
Ever smoker (previous or current)	31 (18%)
Body mass index (kg/m^2^)	24.9 ± 4.4
Physical inactivity^b^	29 (17%)
Alcohol (>1 glass per day)	16 (9%)
*Women-specific factors*	
Age at first menarche	13 ± 1.5
Fertility problems	24 (14%)
Ever pregnant	156 (91%)
– Ever had a miscarriage	53 (34%)
– Children	153 (98%)
– Gestational diabetes	3 (2%)
– Gestational hypertension	32 (21%)
– HELLP syndrome and/or pre-eclampsia	14 (9%)
– Multiparous (≥4 births)	7 (4%)
Polycystic ovary syndrome	3 (2%)
1 or 2 ovaries removed	6 (3%)
Uterus removed	10 (6%)
On hormonal therapy^c^	34 (20%)
Postmenopausal^d^	63 (37%)

Values presented as mean ± SD or *n* (%)

^a^Group comparisons were further assessed based on gender

^b^Not meeting the criterion of a minimum of 30 min of moderately intensive exercise per day

^c^Includes contraceptives and postmenopausal hormonal therapy

^d^Persons older than 55 years were considered postmenopausal

### Psychological factors

Tab. 2 displays data related to psychological factors. Half of the sample reported moderate or high perceived stress in the last month (PSS-10 ≥14).

**Table 2:** Psychological factors of female patients with spontaneous coronary artery dissection (*n* = 172)

Psychological factors	Women (*n* = 172)
*Psychological stress*	
Perceived stress in the last month (PSS-10)	14.7 ± 6.8
– Moderate or high perceived stress (PSS-10, cutoff ≥14)^a^	85 (50%)
Anxiety symptoms (GAD‑7, mean ± SD)	4.9 ± 4.2
– Moderate/severe anxiety (GAD‑7, cutoff ≥10)	21 (12%)
Depressive symptoms (PHQ‑9, mean ± SD)	4.9 ± 3.9
– Moderate/severe depressive symptoms (PHQ‑9 cutoff ≥10)	16 (9%)
Fatigue (FAS-10, mean ± SD)	23.4 ± 6.7
– Substantial or extreme fatigue (FAS-10, cutoff ≥22)	96 (56%)
Sensitivity to physical symptoms (BVS‑3, mean ± SD)	14.3 ± 6.3
Positive mental well-being (MHC-SF, mean ± SD)	3.2 ± 0.8
Burnout	43 (25%)
*Personality characteristics*	
Type D personality (DS14 ≥10 for both NA and SI)	32 (19%)
Neuroticism (BFI-NL neuroticism scale, mean ± SD)	2.8 ± 0.7

*PSS-10* Perceived Stress Scale, 10-item version, *GAD‑7* 7-item General Anxiety Disorder questionnaire, *PHQ‑9* Patient Health Questionnaire, *FAS-10* Fatigue Assessment Scale, *BVS‑3* Body Vigilance Scale, *MHC-SF* Mental Health Continuum-Short Form, *DS14* Type D Scale, *NA* negative affectivity subscale, *SI* social inhibition subscale, *BFI-NL* Dutch version of the Big Five Inventory

^a^Group comparisons were further assessed based on level of perceived stress

With regard to current anxiety levels, 21 women (12%) scored above the cutoff, indicating moderate or severe anxiety symptoms (GAD‑7 ≥10). Moderate or severe depressive symptoms (PHQ‑9 ≥10) were present in 16 women (9%).

More than half of the participants (56%) had substantial or extreme fatigue (FAS-10 ≥22). Approximately one out of five reported a history of burnout. In total, 32 women (19%) scored 10 or higher on both subscales of the DS14, suggesting a Type D personality.

### Comorbidities of SCAD

Tab. 3 shows the presence of comorbid disorders. In total, 130 female SCAD patients were tested for FMD, of whom 38 (29%) screened positive. Mixed connective tissue diseases were present in 3%. More than half reported to have migraine (52%). Other non-cardiovascular chronic conditions were also common, with tinnitus having the highest prevalence (28%). Symptoms of pain were often reported, especially chest pain (68%).

**Table 3:** Comorbidities of female patients with spontaneous coronary artery dissection (*n* = 172)

Comorbidities	Women (*n* = 172)
*No comorbid condition reported*	16 (9%)
*Fibromuscular dysplasia (FMD)*	
Tested on FMD	130 (76%)
– Confirmed diagnosis FMD^a^	38 (29%)
*Other medical conditions*	
Mixed connective tissue diseases (e.g. Marfan syndrome, Ehlers-Danlos syndrome)	5 (3%)
Rheumatoid arthritis	15 (9%)
Vasculitis	4 (2%)
Hypo- or hyperthyroidism	20 (12%)
Allergies	64 (37%)
Migraine^a^	89 (52%)
*Medically chronic symptoms*	
Fibromyalgia	13 (8%)
Chronic fatigue syndrome	9 (5%)
Irritable bowel syndrome	23 (13%)
Tinnitus	48 (28%)
At least one of the above	64 (37%)
*Current pain conditions*	
Chronic pain	50 (29%)
Pain between the shoulder blades	73 (42%)
Stomach pain	39 (23%)
Pain in the jaws or neck	46 (27%)
Chest pain	117 (68%)

^a^Group comparisons were further assessed based on presence of FMD and migraine

### Cluster analyses

Cluster analyses were conducted to determine whether specific SCAD subgroups (clusters) could be identified based on the above-mentioned clinical, psychological, and demographic measures. A three-cluster solution was found with the following main types of patients with SCAD: cluster A (*n* = 40, 23%) was primarily characterised by FMD and chronic non-ischaemic conditions (tinnitus, irritable bowel syndrome, and chronic pain); cluster B (*n* = 65, 38%) was primarily characterised by having migraine; and cluster C (*n* = 67, 39%) consisted of participants without FMD, migraine, or chronic non-ischaemic conditions. The psychological and demographic measures did not contribute to these three clusters.

### Explorative group comparisons

#### FMD and migraine

Consistent with the cluster analysis, we found that female SCAD patients with FMD more often had tinnitus (50% vs 22%, *p* = 0.001), irritable bowel syndrome (24% vs 9%, *p* = 0.041), and chronic fatigue syndrome (16% vs 1%, *p* = 0.003) compared to female patients without FMD. Pain in the jaws or neck was also more common in patients with FMD (39% vs 18%, *p* = 0.015). Furthermore, moderate or severe depressive symptoms (PHQ‑9 ≥10) were more common in patients with FMD compared to those without FMD (16% vs 4%, *p* = 0.034; Electronic Supplementary Material, Table S1a).

Participants who had comorbid migraine (*n* = 89, 52%) more often had elevated anxiety symptoms and fatigue compared to those without migraine (19% vs 5%, *p* = 0.004, and 65% vs 46%, *p* = 0.011, respectively). Positive mental well-being scores were lower in participants with migraine than in those without migraine (*p* = 0.003) (Electronic Supplementary Material, Table S1b).

#### Perceived stress

Moderate or high perceived stress (PSS ≥14) was related to a higher frequency of chronic pain (range 36–52% vs range 17–34%, *p* range 0.002–0.017). A higher perceived stress level was also related to a higher prevalence of other psychological factors, compared to women with a lower stress level (PSS <14) (range 19–82% vs range 0–30%, *p* < 0.001) (Electronic Supplementary Material, Table S1c).

#### Comparison of male versus female participants with SCAD

Men (*n* = 11) had a higher BMI (*p* = 0.023), were younger at the time of the SCAD event (*p* = 0.023), and more often had a part-time or full-time job (*p* < 0.001) compared to women. In contrast, male patients less often had migraine (18% vs 52%, *p* = 0.031), shoulder pain (9% vs 42%, *p* = 0.029), chest pain (36% vs 68%, *p* = 0.046), and chronic pain (0% vs 29%, *p* = 0.037) than did women (Electronic Supplementary Material, Table S2).

## Discussion

This investigation demonstrates that female SCAD patients have high levels of perceived stress and fatigue, whereas levels of anxiety and depression were relatively low. High levels of perceived stress were associated with other psychological measures, but not with medical comorbidities of SCAD such as FMD and migraine. Consistent with previous findings, the prevalence of traditional cardiovascular risk factors was low, except for hypertension. We found three clusters and noted that FMD tended to co-occur with a range of chronic non-ischaemic conditions such as tinnitus, chronic pain, and irritable bowel syndrome. Another cluster (B) consisted of patients characterised by having migraine. The present findings indicate that psychological factors, in addition to FMD and migraine, may contribute to the development of SCAD and to the subsequent quality of life and clinical course of patients after SCAD.

### Comparison with previous studies

The characteristics of the participants are comparable to those of other large published observational cohorts, in terms of the high percentage of women (94%), and the low mean age compared to patients with ischaemic heart disease (IHD) [4, 5, 20]. Half of the sample reported a history of migraine (52%), which is markedly higher than prevalence estimates in the general Dutch population (17% in people aged between 45 and 60 years) [21]. The presence of FMD was consistent with that in prior studies in SCAD patients, with 29% having a confirmed FMD diagnosis [3, 4, 20, 22]. In our study, we did not distinguish between the diagnostic features of FMD, and not all patients were screened for FMD.

The observed anxiety (12%) and depression (9%) levels in our sample are comparable to those of women in the general Dutch population of similar age (12 and 7%, respectively) [23, 24], but lower than what has been found in women with typical IHD, in whom the prevalence estimates of anxiety and depression are 29 and 39%, respectively [25, 26]. It is possible that our findings reflect the higher percentages of married, highly educated, and employed participants in our study sample [27]. The high level of burnout prior to SCAD (20%) is noteworthy and slightly higher than population-based prevalence estimates (17%) [28].

### Limitations

Several study limitations should be mentioned. Since the major part included highly educated women who registered themselves for the Dutch SCAD registry and actively asked for a second opinion, referral bias and sampling bias might have influenced our results. However, since other studies and review papers did not report the education level of patients with clinical or psychological measures, future studies should investigate the role of education level in SCAD patients.

Another limitation is that the comorbidities, other than FMD, were documented based on participants’ self-report, which might have led to an overestimation of the prevalence of these conditions. However, the prevalence of migraine and FMD was similar to that in prior reports in SCAD patients [3, 4, 20, 22].

The present data suggest there may be important sex differences in the experienced symptoms after SCAD. However, the low number of men might have caused a lack of power in our group comparisons by sex, and these data need validating in a larger cohort. In addition, the cross-sectional design precludes conclusions that can be drawn from this study regarding causal pathways. Longitudinal studies are needed to better identify risk factors for incident SCAD and to investigate variations in psychological consequences of SCAD over time.

### Implications for future research and clinical practice

High stress levels in SCAD patients might be caused by the rarity of the disease, the unclear pathogenesis, and uncertain optimal management [5]. The lack of informational support may affect stress symptoms and coping strategies [29]. It will therefore be important to educate health professionals regarding the predictors and consequences of SCAD. In addition, accurate information should be made available for SCAD patients [29]. Tailored cardiac rehabilitation programmes focusing on exercise and psychological factors might be helpful for improvements in both physical and emotional domains [30].

## Conclusion

High levels of perceived stress and fatigue are common in female SCAD patients. Furthermore, several (chronic) pain conditions, tinnitus, and burnout were frequently reported precursors or comorbidities of SCAD, in addition to well-documented comorbidities of SCAD (i.e. FMD and migraine). Our findings may contribute to the development of tailored rehabilitation and prevention programmes for persons at elevated SCAD risk. Greater focus on long-lasting psychological and symptomatic consequences in the care and rehabilitation of SCAD patients has the potential to significantly improve longer-term patient morbidity and improve quality of life.

## Caption Electronic Supplementary Material

Table S1a. Explorative group comparisons: FMD vs. no FMD. Table S1b. Explorative group comparisons: Migraine vs. no migraine. Table S1c. Explorative group comparisons: Moderate or high stress vs. no stress. Table S2. Explorative group comparisons: Women vs. men
