# Supplementary material for: Psychological and clinical characteristics of female patients with spontaneous coronary artery dissection
Source: Neth Heart J. 2020 Jun 4;28(9):485–91. doi: 10.1007/s12471-020-01437-7 (PMC7431500; doi:10.1007/s12471-020-01437-7)
Supplement: Supplementary file 1 — Table S1a. Explorative group comparisons: FMD vs. no FMD. Table S1b. Explorative group comparisons: Migraine vs. no migraine. Table S1c. Explorative group comparisons: Moderate or high stress vs. no stress. Table S2. Explorative group comparisons: Women vs. men [file 12471_2020_1437_MOESM1_ESM.pdf]

SUPPLEMENTAL FILES

Supplemental Table S1. Explorative group comparisons

Table S1a. Explorative group comparisons: FMD vs. no FMD

	No FMD (n=92)	FMD (n=38)	Fisher's exact test/Chi-square/Anova
Irritable bowel syndrome	8 (9%)	9 (24%)	FET, *p*=0.041
Tinnitus	20 (22%)	19 (50%)	$X^2(1) = 10.23$, *p*=0.001
Chronic fatigue syndrome	1 (1%)	6 (16%)	FET, *p*=0.003
Pain in jaws or neck	17 (18%)	15 (39%)	$X^2(1) = 6.39$, *p*=0.015
Moderate or severe depressive symptoms (PHQ-9≥10)	4 (4%)	6 (16%)	FET, *p*=0.034

Table S1b. Explorative group comparisons: Migraine vs. no migraine

	No migraine (n=83)	Migraine (n=89)	Fisher's exact test/Chi-square/Anova
Hypertension	18 (22%)	36 (40%)	$X^2(1) = 7.02$, *p*=0.008
Rheumatic disorder	2 (2%)	13 (15%)	$X^2(1) = 8.03$, *p*=0.005
Tinnitus	17 (20%)	31 (35%)	$X^2(1) = 4.40$, *p*=0.042
Hypo-or hyperthyroidism	5 (6%)	15 (17%)	$X^2(1) = 4.90$, *p*=0.027
Chronic pain	15 (18%)	35 (39%)	$X^2(1) = 9.41$, *p*=0.002
Other (hormonal) disorder	2 (2%)	9 (10%)	$X^2(1) = 4.26$, *p*=0.039
No clinical history of comorbidities	16 (19%)	0	$X^2(1) = 18.9$, *p*<0.001

Moderate or severe anxiety symptoms (GAD7≥10)	4 (5%)	17 (19%)	$X^2(1) = 8.34$, $p=0.004$
Substantial or extreme fatigue (FAS-10≥22)	38 (46%)	58 (65%)	$X^2(1) = 6.54$, $p=0.011$
Positive well-being (MHC-SF, mean±SD)	3.4±0.8	3.0±0.9	$F(1,169)=8.83$, $p=0.003$

Table S1c. Explorative group comparisons: Moderate or high stress vs. no stress

	**No stress (PSS<14) (n=86)**	**Moderate or high stress (PSS≥14) (n=85)**	**Fisher's exact test/Chi-square/Anova**
Pain between the shoulder blades	29 (34%)	44 (52%)	$X^2(1) = 5.69$, $p=0.017$
Pain in jaws or neck	15 (17%)	31 (36%)	$X^2(1) = 7.87$, $p=0.005$
Chest pain	49 (57%)	67 (79%)	$X^2(1) = 9.35$, $p=0.002$
Moderate or severe anxiety symptoms (GAD7≥10)	1 (1%)	20 (24%)	$X^2(1) = 20.13$, $p<0.001$
Moderate or severe depressive symptoms (PHQ-9≥10)	0	16 (19%)	$X^2(1) = 17.88$, $p<0.001$
Substantial or extreme fatigue (FAS-10≥22)	26 (30%)	70 (82%)	$X^2(1) = 47.16$, $p<0.001$
Type D personality (DS14≥10)	4 (5%)	28 (33%)	$X^2(1) = 22.49$, $p<0.001$
Neuroticism (BFI-NL neuroticism scale, mean±SD)	2.4±0.6	3.2±0.6	F(1,169)=87.36, $p<0.001$
Positive well-being (MHC-SF, mean±SD)	3.5±0.7	2.9±0.8	F(1,168)=29.59, $p<0.001$

Table S2. Explorative group comparisons: Women vs. men

	**Women (n=172)**	**Men (n=11)**	**Fisher's exact test/Chi-square/Anova**
Body Mass Index (BMI)	24.9±4.4	28.4±10.3	$F(1,180)=5.23$, $p=0.023$
Age at most recent SCAD event	49.4±7.6	43.9±7.9	$F(1,181)=5.27$, $p=0.023$
Working full time or part time	129 (75%)	11 (100%)	FET, $p<0.001$
Migraine	89 (52%)	2 (18%)	$X^2(1) = 4.66$, $p=0.031$
Pain between the shoulder blades	73 (42%)	1 (9%)	FET, $p=0.029$
Chest pain	117 (68%)	4 (36%)	FET, $p=0.046$
Chronic pain	50 (29%)	0	FET, $p=0.037$
